# Serum Magnesium is Inversely Associated with Body Composition and Metabolic Syndrome

**DOI:** 10.2147/DMSO.S391369

**Published:** 2023-01-12

**Authors:** AlMaha Al Shammaa, Amna Al-Thani, Maryam Al-Kaabi, Kaltham Al-Saeed, Maria Alanazi, Zumin Shi

**Affiliations:** 1 Human Nutrition Department, College of Health Sciences, QU Health, Qatar University, Doha, Qatar

**Keywords:** serum magnesium, DEXA, body adiposity, total fat mass, Qatar Biobank, cross-sectional study

## Abstract

**Purpose:**

Magnesium is vital to maintain normal physiological functions. We aimed to identify the association between serum magnesium and different measures of body adiposity among Qatari adults. We hypothesized that the association was mediated by depression and sleep duration.

**Patients and Methods:**

The study included 1000 adults aged 20 years and above who attended the Qatar Biobank Study (QBB) between 2012 and 2019. Body adiposity was assessed using dual-energy X-ray absorptiometry (DEXA). Serum magnesium concentration was measured. Sub-optimal magnesium was defined as magnesium concentration less than 0.85 mmol/L. The association was examined using linear regression.

**Results:**

The mean age of the participants (n=1000) was 35.8 (SD 10.3) years. More than half of the participants had sub-optimal magnesium concentrations (60.2% in men and 52.3% in women). Serum magnesium was inversely associated with different types of fat mass. In the fully adjusted model, per 1 SD increment of serum magnesium had standardized regression coefficients of −0.09 (p 0.005) for total fat mass, −0.08 (p 0.008) for trunk fat, −0.09 (p 0.003) for gynoid fat and −0.08 (p 0.008) for android fat. There was no gender difference in the association. The inverse association between serum magnesium and fat mass was significant in those with sleep duration ≥7 hours but not in those <7 hours. Depressive symptom and sleep did not mediate the association between serum magnesium and fat mass. Serum magnesium was inversely associated with metabolic syndrome (per 1 SD increment had an odds ratio (OR) of 0.70 (95% CI 0.57–0.85)).

**Conclusion:**

There was an inverse association between serum magnesium and fat mass, especially among those with an adequate sleep duration and without chronic conditions including diabetes, hypertension and depression.

## Introduction

Magnesium is a crucially important metal in the human body. As a cofactor of more than 300 enzymes, magnesium is involved in various physiological activities including protein synthesis, blood pressure regulation, glucose homeostasis, and regulation of nerve and muscle function.^1–3^ Serum magnesium level is considered a poor indicator of magnesium status as the majority is stored in the bones. Total magnesium found in the body is typically around 25g with 50–60% contained in the bones and soft tissue with the blood serum only holding 1%.^4^ Magnesium deficiency is common due to health problems and inadequate dietary intake, and largely goes undiagnosed.^5^ Inadequate magnesium level induces chronic low-grade inflammation and may cause many health problems including obesity,^6^ cardiovascular diseases,^7^ diabetes^8^,^9^, depressive symptoms^10^ as well as sleep disorders.^11^ Most of the existing population studies on the association between magnesium status and obesity were based on the use of body mass index (BMI) or waist circumference to define obesity.^12–15^ There is a limited number of studies using direct measures of different types of adiposity.^16^,^17^ The use of BMI to estimate body fat has its limitations.^18^ For example, for a given BMI, the body fat percentage changes with age, sex and ethnicity.^18^ Furthermore, BMI cannot differentiate fat mass from muscle mass. Body fat measured by DEXA is regarded as the gold standard in clinical settings. It can measure site specific fat content. Based on the literature, the accumulation of visceral fat, rather than subcutaneous fat, is associated with increased cardiometabolic risk.^19^ However, the use of DEXA in large epidemiological studies is limited due to high cost.

As short sleep duration^20^ and depression^21^ are positively associated with obesity, the association between magnesium and obesity may be mediated by sleep and depression. However, no study has tested the mediation effect of sleep and depression.

Magnesium-rich food includes green vegetables, nuts, seeds and whole grains. Globally, high consumption of refined grains and low intake of vegetables are believed to be important causes of inadequate intake of magnesium.^5^ Furthermore, during past decades, the soil used for agriculture is becoming deficient in magnesium. As a result, the concentration of magnesium in fruit and vegetables has decreased by 20–30%.^2^ Another significant contributor to inadequate magnesium intake is the low level of magnesium in drinking water, which is often caused by the use of desalinated seawater.^22^,^23^ It has been reported that about two-thirds of the adults in western countries do not consume the estimated average requirement for magnesium.^2^

Qatar is one of the countries with the highest burden of obesity in the world. In a national survey conducted in 2012, the prevalence of obesity was 41% among adults aged 18–64 years.^24^ High consumption of refined grains combined with the dependence of desalinated seawater predisposes Qatari to magnesium deficiency.^22^,^23^ In a previous study in Qatari adults, it was found that the prevalence of inadequate serum magnesium was more than 60%.^8^ Thus, Qatar provides a great opportunity to examine the association between serum magnesium and obesity. Using data from the Qatar Biobank Study (QBB), the aim of the study was to 1) assess the association between serum magnesium and body adiposity assessed by dual-energy x-ray absorptiometry (DEXA); and 2) examine the mediation effect of sleep and depression symptom on the association. We hypothesized that sleep and depression mediate the association between serum magnesium and body adiposity. In order to assess the clinical implications of the association between serum magnesium and body adiposity, we further examined the association between serum magnesium and metabolic syndrome.

## Materials and Methods

### Study Population

The QBB study started in 2012 and recruited adults aged 18 years and above who were Qatari or who had lived in Qatar for more than 15 years. The details of the QBB study have been published elsewhere.^25^ Data on sociodemographic information, lifestyle factors, and dietary habits were collected using a self-administered questionnaire. Information on health conditions and medication use was collected through nurse interviews. Each participant underwent a health examination at Hamad Medical Center, Doha.

For the current analysis, we included a random sample of 1000 adults aged 20 years and above who had data on serum magnesium and underwent DEXA measures. The sample size was determined by the fact that QBB provides 1000 samples to research projects conducted at Qatar University for free. The ethical approval of the Qatar Biobank study was provided by the Institutional Review Board from the Hamad Medical Corporation Ethics Committee in Qatar. The current study was approved by the Institutional Review Board of Qatar Biobank (Ex-2021-QF-QBB-RES-ACC-0048-0165) and was carried out in accordance with the Declaration of Helsinki, as revised in 2008. Qatar Biobank provided the access to the data used in the study. Each participant gave a written informed consent.

### Outcome Variable: Body Adiposity (Total Fat Mass, Gynoid Fat Mass, Trunk Fat Mass, and Android Fat Mass)

Body adiposity was measured using Lunar iDXA (SN 210520, GE Healthcare, USA). The following measures were included in the analysis as the outcome variables: total fat mass, gynoid fat mass, trunk fat mass, and android fat mass. The android region was defined as the area between the ribs and the pelvis. The gynoid region was defined as the area of the hips and the upper thighs, and it overlapped the leg and trunk areas.

Metabolic syndrome was defined based on the literature.^26^ An individual was categorized as having metabolic syndrome if he/she had ≥3 of the following components: 1) elevated waist circumference (≥102 cm in men, ≥88 cm in women); 2) high blood pressure (Systolic ≥130 and/or diastolic ≥85 mm Hg) or drug treatment for hypertension; 3) low HDL-cholesterol (<40 mg/dl in men and <50 mg/dl in women) or drug treatment for dyslipidemia; 4) high TG (≥150 mg/dL) or drug treatment for dyslipidemia; 5) Elevated fasting glucose (≥100 mg/dL, drug treatment of elevated glucose is an alternate indicator).

### Exposure Variable: Serum Magnesium

Serum magnesium was measured using an automated colorimetric method (Magnesium Gen.2 from Roche Diagnostics, Indianapolis, IN). The measurement was conducted in a central laboratory of the Qatar Biobank Study and the assay’s coefficients of variations are 0.3% to 0.8%. In our analysis, subclinical magnesium deficiency was defined as serum magnesium <0.85 mmol/L.^5^

### Covariates

In the analyses, socioeconomic status and lifestyle factors were treated as covariates. They included education level (low: primary and secondary school; medium: Technical/professional school; high: graduate and post-graduate degree), gender, age, smoking (current smoker, non-smoker, ex-smoker), leisure time physical activity level (metabolic equivalent of task (MET), recoded as tertiles), and BMI (overweight: 25–29.9 kg/m^2^ and obesity: ≥30 kg/m^2^). Sleep duration was self-reported. Hypertension was defined as systolic blood pressure ≥140 mmHg and/or diastolic blood pressure ≥90 mmHg or a self-reported previous diagnosis of hypertension by the physician.^27^ Participants were also asked about medication use for hypertension. Diabetes was defined as glycated hemoglobin (HbA1c) ≥6.5%, random blood glucose ≥11.1 mmol/l, fasting blood glucose ≥7 mmol/l, or self-reported diabetes.^28^ Kidney function was evaluated by the estimated glomerular filtration rate (eGFR). eGFR was calculated by the adaptation of the CKD-EPI creatinine equation.^29^ Total cholesterol, HDL-C and triglyceride levels were measured using standard laboratory enzymatic methods. While LDL-C was calculated using the Friedewald formula.^30^ C-reactive protein was used as an indicator of inflammation and recoded into <6 mg/L or ≥6 mg/L. Depressive symptoms were assessed using the Patient Health Questionnaire (PHQ-9).^31^

Habitual food frequency intake was assessed by a food frequency questionnaire (FFQ). The tool was developed based on the FFQ used in the European Prospective Investigation of Cancer (EPIC) study and included 102 commonly consumed food items in Qatar. However, the FFQ has not been validated in Qatar. Dietary patterns were constructed using factor analysis based on a previous study in Qatar.^32^ Three dietary patterns were identified. Factor loadings of the identified dietary patterns were presented in Supplement Table 1. In the analyses, dietary patterns were treated as potential confounding factors.

### Statistical Analyses

Serum magnesium concentrations were categorized into quartiles. Sample characteristics were presented as mean (SD) or percentage. ANOVA and chi-square test were used to test the difference in continuous or categorical variables by quartiles of serum magnesium. Multivariable linear regression models were used to assess the association between body adiposity and serum magnesium. The multivariable linear regression model was adjusted for age, gender, education, smoking, physical activity, and dietary patterns. Structure equation model was used to test whether depressive symptom and sleep mediate the effect of magnesium on body adiposity.

To test the non-linear association between serum magnesium and body adiposity, the restricted cubic spline method was used. Three knots were put at 10, 50 and 90 percentiles. In this study, *p*-values < 0.05 were considered statistically significant. All the analyses were conducted using STATA (Version 17, STATA Corporation, College Station, TX, USA).

## Results

### Sample Description

The mean ±SD age of the 1000 participants was 35.8±10.3 years. The mean serum magnesium was 0.84±0.08 mmol/L. Overall, 56.3% had serum magnesium below 0.85 mmol/dl (60.2% in women and 52.3% in men). More than half of the participants were highly educated and 4.8% had low education levels. A total of 17.8% were current smokers. Leisure-time physical activity was low. Most of the participants were overweight (38.8%) or obese (31.4%). A CRP level ≥ 6 mg/L was found in 22.1% of the participants. More than 60% of the participants reported using dietary supplements.

Across the quartiles of serum magnesium levels from low to high, there were no differences in age, education, sleep duration, smoking and PHQ-9 score (Table 1). BMI decreased with the increase of serum magnesium. Vitamin D and calcium use were more common among those with low serum magnesium. However, no difference in fruit and vegetable intake was found across the quartiles of serum magnesium.

**Table 1:** Sample Characteristics by Quartiles of Serum Magnesium Among Participants Attending Qatar Biobank Study (N=1000)

	Q1	Q2	Q3	Q4	*P* value
	N=303	N=260	N=203	N=234	
Magnesium (mmol/L)	0.77(0.03)	0.83 (0.01)	0.86 (0.01)	0.91 (0.03)	<0.001
Age	36.1 (10.4)	35.7 (10.9)	36.6 (10.5)	36.2 (10.4)	0.817
Sex					<0.001
Male	121 (39.9%)	137 (52.7%)	98 (48.3%)	137 (58.5%)	
Female	182 (60.1%)	123 (47.3%)	105 (51.7%)	97 (41.5%)	
Education					0.700
Low	18 (5.9%)	13 (5.0%)	11 (5.4%)	6 (2.6%)	
Medium	83 (27.4%)	71 (27.4%)	57 (28.1%)	70 (30.0%)	
High	202 (66.7%)	175 (67.6%)	135 (66.5%)	157 (67.4%)	
Smoking					0.072
Non	221 (72.9%)	168 (64.6%)	144 (70.9%)	153 (65.4%)	
Smoker	42 (13.9%)	47 (18.1%)	36 (17.7%)	53 (22.6%)	
Ex-smoker	40 (13.2%)	45 (17.3%)	23 (11.3%)	28 (12.0%)	
Leisure time physical activity (MET hours/week)	6.3 (30.0)	6.3 (17.7)	6.0 (20.1)	5.4 (17.1)	0.967
BMI (kg/m^2^)	28.6 (5.6)	28.5 (6.2)	27.9 (5.9)	27.5 (5.1)	0.090
BMI categories					0.131
Normal	82 (27.1%)	75 (28.8%)	72 (35.5%)	69 (29.5%)	
Overweight	119 (39.3%)	93 (35.8%)	73 (36.0%)	103 (44.0%)	
Obese	102 (33.7%)	92 (35.4%)	58 (28.6%)	62 (26.5%)	
Cholesterol Total (mmol/l)	4.8 (0.9)	4.9 (0.9)	5.1 (1.0)	5.0 (1.0)	0.007
HDL-Cholesterol(mmol/l)	1.4 (0.4)	1.4 (0.4)	1.4 (0.4)	1.3 (0.3)	0.018
Triglyceride (mmol/l)	1.3 (0.8)	1.4 (1.2)	1.3 (0.8)	1.4 (0.9)	0.213
LDL-Cholesterol (mmol/l)	2.9 (0.8)	2.9 (0.8)	3.1 (0.9)	3.1 (0.9)	0.001
Supplement use	200 (66.0%)	158 (60.8%)	125 (61.6%)	136 (58.1%)	0.292
Vitamin D and calcium use	136 (44.9%)	99 (38.1%)	76 (37.4%)	75 (32.1%)	0.024
Vegetable intake (times/week)	17.9 (14.4)	17.4 (14.9)	15.4 (12.0)	17.3 (13.2)	0.250
Fruit intake (times/week)	7.2 (6.5)	6.6 (6.2)	7.2 (5.8)	6.3 (5.9)	0.310
Dietary pattern 1	0.05 (1.15)	−0.03 (0.93)	−0.03 (0.93)	−0.01 (0.93)	0.711
Dietary pattern 2	0.03 (1.03)	0.01 (1.02)	0.00 (0.96)	−0.05 (0.98)	0.800
Dietary pattern 3	0.06 (1.09)	0.05 (1.01)	−0.01 (1.01)	−0.11 (0.85)	0.220
eGFR (mL/min/1.73m^2^)	113.5 (14.3)	111.8 (13.6)	110.7 (14.0)	109.2 (14.0)	0.003
HbA1C (%)	5.7 (1.2)	5.5 (0.8)	5.5 (0.7)	5.5 (0.6)	0.010
Hypertension	39 (12.9%)	21 (8.1%)	21 (10.3%)	21 (9.0%)	0.260
Diabetes	48 (16.4%)	29 (11.5%)	17 (8.9%)	25 (11.2%)	0.072
Metabolic syndrome	61 (20.1%)	33 (12.7%)	27 (13.3%)	31 (13.2%)	0.041
Insulin use	13 (4.3%)	5 (1.9%)	2 (1.0%)	0 (0.0%)	0.003
Diabetes medication other than insulin	30 (9.9%)	12 (4.6%)	8 (3.9%)	8 (3.4%)	0.003
Hypertension medication use	31 (10.2%)	11 (4.2%)	7 (3.4%)	9 (3.8%)	0.001
PHQ-9 score	6.6 (4.8)	6.4 (4.6)	6.0 (4.6)	5.8 (4.4)	0.160
Sleep duration <7 hours	164 (54.5%)	131 (50.4%)	119 (58.9%)	128 (56.9%)	0.280
CRP ≥6 (mg/L)	67 (22.1%)	46 (17.7%)	41 (20.2%)	47 (20.1%)	0.490
Total fat mass (kg)	31.5 (11.2)	30.5 (11.7)	29.6 (11.2)	29.0 (10.3)	0.055
Trunk fat (kg)	16.0 (6.6)	15.7 (6.8)	15.2 (6.6)	15.1 (6.3)	0.420
Gynoid fat mass (kg)	5.4 (1.8)	5.2 (2.0)	5.0 (1.9)	4.8 (1.7)	0.003
Android fat mass (kg)	2.6 (1.3)	2.6 (1.3)	2.5 (1.3)	2.5 (1.2)	0.560

**Notes**: Data are presented as mean (SD) for continuous measures, and n (%) for categorical measures.

**Abbreviations**: CRP, c-reactive protein; PHQ-9, Patient Health Questionnaire.

Age, CRP levels, PHQ-9 score and gender were associated with most types of fat mass (Supplement Figure 1). Compared with those with CRP<6, CRP≥6 mg/L was associated with 9.2 kg higher total fat mass. However, sleeping less than 7 hours was not associated with fat mass.

### Association Between Serum Magnesium and Fat Mass

Serum magnesium was inversely associated with different types of fat mass. Across the quartiles of serum magnesium, the mean total fat mass decreased gradually from 31.5 kg to 29.0 kg. A similar trend was seen for gynoid fat mass (from 5.4 kg to 4.8 kg). In the fully adjusted model, for 1 SD increase of serum magnesium, the regression coefficients (95% CI) were −0.98 (95% CI −1.66 to −0.30, p value =0.005) for total fat, −0.53 (95% CI −0.93 to −0.14, p value = 0.008) for trunk fat, −0.17 (95% CI −0.29 to −0.06, p value =0.003) for gynoid fat and −0.10 (95% CI −0.18 to −0.03, p value = 0.008) for android fat (Table 2). The standardized regression coefficients associated with 1 SD increment of serum magnesium were −0.09 (p 0.005) for total fat mass, −0.08 (p 0.008) for trunk fat, −0.09 (p 0.003) for gynoid fat and −0.08 (p 0.008) for android fat. The associations were not materially changed after further adjusting for kidney function, or medication use for hypertension and diabetes (data not shown).

**Table 2:** Association Between Serum Magnesium and Fat Mass Among Qatari Adults (N=1000)

	Quartiles of Serum Magnesium				*p* -value for Trend	Serum Magnesium (Continuous, Per SD)	
	Q1	Q2	Q3	Q4			*p* -value
Total fat mass							
Unadjusted	0.00	−0.94 (−2.78,0.91)	−1.84 (−3.82,0.14)	**−2.52 (−4.41,-0.62)**	0.006	**−1.33 (−2.02, −0.65)**	<0.001
Adjusted *	0.00	−0.34 (−2.14,1.46)	−1.53 (−3.45,0.40)	−1.65 (−3.52,0.22)	0.044	**−0.98 (−1.66, −0.30)**	0.005
Trunk fat mass							
Unadjusted	0.00	−0.27 (−1.37, 0.82)	−0.78 (−1.96, 0.39)	−0.82 (−1.94, 0.31)	0.105	**−0.59 (−1.00, −0.18)**	0.004
Adjusted *	0.00	−0.21 (−1.26, 0.83)	−0.82 (−1.94, 0.29)	−0.80 (−1.88, 0.29)	0.089	**−0.53 (−0.93, −0.14)**	0.008
Gynoid fat mass							
Unadjusted	0.00	−0.19 (−0.50, 0.13)	−0.39 (−0.73, −0.06)	−0.58 (−0.91, −0.26)	<0.001	**−0.26 (−0.37, −0.14)**	<0.001
Adjusted *	0.00	−0.03 (−0.34, 0.27)	−0.29 (−0.61, 0.03)	**−0.34 (−0.65, −0.03)**	0.013	**−0.17 (−0.29, −0.06)**	0.003
Android fat mass							
Unadjusted	0.00	−0.05 (−0.26, 0.16)	−0.14 (−0.36, 0.09)	−0.13 (−0.35, 0.09)	0.171	**−0.11 (−0.19, −0.03)**	0.007
Adjusted *	0.00	−0.05 (−0.25, 0.15)	−0.15 (−0.37, 0.06)	−0.15 (−0.35, 0.06)	0.106	**−0.10 (−0.18, −0.03)**	0.008

**Notes**: Values are regression coefficients (95% CI). Bold fonts represent statistically significant regression coefficients. *Model adjusted for age, gender, education, smoking, physical activity, and dietary patterns.

### Subgroup Analyses

In subgroup analyses, we did not find any significant interaction between serum magnesium with gender, diabetes, hypertension, smoking, PHQ-9, sleep duration, CRP, and supplement use (Table 3). However, the association between serum magnesium and total fat mass was stronger among those with sleep duration ≥7 hours as compared with those <7 hours (p for interaction 0.108). The regression coefficients were −1.60 (95% CI −2.71 to −0.49) and −0.47 (95% CI −1.34 to 0.39) among those with and without sleep≥7 hours, respectively. Similar patterns were also observed for the interaction between sleep and serum magnesium in relation to all other adiposity measures.

**Table 3:** Subgroup Analyses of the Association Between Serum Magnesium and Fat Mass

	Total Fat Mass		Trunk Fat		Gynoid Fat		Android Fat	
	β (95% CI)	p*	β (95% CI)	p*	β (95% CI)	p*	β (95% CI)	p*
Gender		0.654		0.468		0.491		0.477
Male	**−1.20 (−2.23, −0.17)**		**−0.71 (−1.34, −0.09)**		**−0.22 (−0.39, −0.05)**		**−0.14 (−0.26, −0.02)**	
Female	−0.81 (−1.70, 0.08)		−0.39 (−0.89, 0.10)		−0.13 (−0.29, 0.02)		−0.08 (−0.17, 0.01)	
Diabetes		0.145		0.493		0.031		0.480
No	**−1.22 (−1.98, −0.46)**		**−0.56 (−0.99, −0.12)**		**−0.24 (−0.37, −0.11)**		**−0.11 (−0.19, −0.02)**	
Yes	0.28 (−1.44, 1.99)		−0.12 (−1.22, 0.98)		0.08 (−0.18, 0.34)		−0.03 (−0.23, 0.18)	
Hypertension		0.147		0.237		0.205		0.235
No	**−0.75 (−1.47, −0.03)**		−0.41 (−0.83, 0.00)		**−0.14 (−0.26, −0.02)**		−0.08 (−0.16, 0.00)	
Yes	−2.08 (−4.18, 0.02)		−1.05 (−2.24, 0.15)		**−0.36 (−0.69, −0.04)**		−0.22 (−0.44, 0.01)	
Smoking		0.570		0.848		0.460		0.903
None	**−1.19 (−1.99, −0.39)**		**−0.60 (−1.06, −0.14)**		**−0.22 (−0.35, −0.08)**		**−0.11 (−0.20, −0.03)**	
Smoker	−0.87 (−2.54, 0.79)		−0.48 (−1.48, 0.52)		−0.12 (−0.39, 0.15)		−0.10 (−0.29, 0.10)	
Ex-smoker	−0.59 (−2.80, 1.62)		−0.47 (−1.82, 0.88)		−0.10 (−0.46, 0.25)		−0.10 (−0.37, 0.16)	
Sleep duration		0.108		0.101		0.111		0.157
<7 hrs	−0.47 (−1.34, 0.39)		−0.22 (−0.73, 0.29)		−0.09 (−0.24, 0.05)		−0.05 (−0.15, 0.05)	
≥7 hrs	**−1.60 (−2.71, −0.49)**		**−0.89 (−1.52, −0.26)**		**−0.28 (−0.47, −0.09)**		**−0.16 (−0.28, −0.04)**	
PHQ-9 score		0.931		0.547		0.415		0.518
<10	**−0.96 (−1.66, −0.26)**		**−0.48 (−0.88, −0.08)**		**−0.19 (−0.31, −0.08)**		**−0.09 (−0.17, −0.01)**	
≥10	−0.64 (−2.71, 1.42)		−0.51 (−1.76, 0.74)		−0.04 (−0.39, 0.32)		−0.11 (−0.34, 0.13)	
CRP		0.804		0.960		0.867		0.682
<6 mg/L	**−1.07 (−1.77, −0.37)**		**−0.57 (−0.98, −0.17)**		**−0.20 (−0.32, −0.08)**		**−0.12 (−0.20, −0.04)**	
≥6 mg/L	−1.29 (−3.00, 0.42)		−0.64 (−1.64, 0.36)		−0.18 (−0.46, 0.11)		−0.09 (−0.28, 0.09)	
Supplement use		0.921		0.711		0.741		0.688
No	−0.99 (−2.14, 0.15)		−0.46 (−1.13, 0.22)		**−0.20 (−0.40, −0.01)**		−0.09 (−0.22, 0.04)	
Yes	−0.83 (−1.68, 0.02)		**−0.50 (−1.00, −0.01)**		−0.14 (−0.28, 0.01)		**−0.10 (−0.19, −0.01)**	

**Notes**:*p for interaction. Model adjusted for age, gender, education, smoking, physical activity, and dietary patterns. Stratification variables were not adjusted in corresponding models. Values represent regression coefficients (95% CI) for per SD increase of serum magnesium. Bold fonts represent statistically significant regression coefficients (95% CI).

### Non-Linear Association and Mediation

In restricted cubic spline regression, there were no significant non-linear associations between serum magnesium and fat mass. All the associations were linear. Sleep duration and depressive symptom were not mediators of the association between serum magnesium and all adiposity measures (data not shown).

### Association Between Serum Magnesium and Metabolic Syndrome

Serum magnesium was inversely associated with metabolic syndrome (Table 4). In the fully adjusted model, for 1 SD increase of serum magnesium, the odds ratio (OR) for metabolic syndrome was 0.70 (95% CI 0.57–0.85). Furthermore, serum magnesium was inversely associated with components of metabolic syndrome, including elevated TG (OR 0.79 (0.68–0.93)) and elevated glucose (OR 0.74 (0.61–0.89)).

**Table 4:** Association Between Serum Magnesium and Metabolic Syndrome Among Qatari Adults (N=1000)

	Quartiles of Serum Magnesium					Serum Magnesium (Continuous, Per SD)	
	Q1	Q2	Q3	Q4	P for Trend		P value
Metabolic syndrome							
Unadjusted	1.00	**0.58 (0.36–0.91)**	**0.61 (0.37–1.00)**	**0.61 (0.38–0.97)**	0.032	**0.70 (0.59–0.84)**	<0.001
Adjusted	1.00	**0.52 (0.30–0.89)**	**0.53 (0.30–0.94)**	**0.55 (0.31–0.96)**	0.030	**0.70 (0.57–0.85)**	<0.001
Elevated blood pressure							
Unadjusted	1.00	0.69 (0.44–1.08)	0.86 (0.54–1.37)	0.83 (0.53–1.29)	0.529	0.93 (0.79–1.10)	0.379
Adjusted	1.00	0.61 (0.36–1.01)	0.78 (0.46–1.31)	0.70 (0.42–1.18)	0.267	0.93 (0.77–1.12)	0.433
Reduced HDL							
Unadjusted	1.00	0.66 (0.45–0.95)	0.84 (0.57–1.23)	0.91 (0.63–1.32)	0.791	0.92 (0.81–1.06)	0.261
Adjusted	1.00	0.67 (0.46–0.98)	0.83 (0.56–1.23)	0.90 (0.61–1.32)	0.723	0.92 (0.80–1.06)	0.265
Elevated TG							
Unadjusted	1.00	0.76 (0.53–1.10)	**0.63 (0.42–0.95)**	0.87 (0.60–1.26)	0.268	**0.84 (0.73–0.96)**	0.013
Adjusted	1.00	0.68 (0.45–1.02)	**0.53 (0.34–0.83)**	0.73 (0.48–1.11)	0.067	**0.79 (0.68–0.93)**	0.004
Elevated glucose							
Unadjusted	1.00	0.68 (0.44–1.06)	**0.51 (0.30–0.85)**	0.70 (0.44–1.10)	0.051	**0.73 (0.61–0.87)**	<0.001
Adjusted	1.00	0.67 (0.41–1.10)	**0.48 (0.27–0.84)**	0.68 (0.41–1.14)	0.055	**0.74 (0.61–0.89)**	0.002
Central obesity							
Unadjusted	1.00	0.97 (0.66–1.42)	0.92 (0.60–1.39)	0.80 (0.53–1.21)	0.287	**0.86 (0.74–0.99)**	0.042
Adjusted	1.00	1.06 (0.70–1.61)	0.94 (0.60–1.48)	0.92 (0.59–1.43)	0.630	0.92 (0.79–1.08)	0.293

**Notes**: Model adjusted for age, gender, education, smoking, physical activity, and dietary patterns. Values represent odds ratio (95% CI). Bold fonts represent statistically significant odds ratios (95% CI).

## Discussion

In this cross-sectional study, there was a significant inverse association between serum magnesium and fat mass including total, android, trunk, gynoid fat mass as well as metabolic syndrome. Low serum magnesium was prevalent among Qatari adults accompanied by increased obesity rates and BMI values. The difference in total fat mass was 2.5 kg comparing extreme quartiles of serum magnesium. Finding from this study did not support our hypothesis that short sleep and depression are mediators of the association between serum magnesium and body fat mass.

The high prevalence of sub-optimal serum magnesium levels in our sample could be explained by several factors including a high consumption of refined grains and fast food, inadequate intake of vegetables, fruits, and whole grains^5^,^8^,^33^ as well as the use of desalinated water as drinking water.^22^ Furthermore, the high prevalence of vitamin D deficiency (90.4%) in Qatar may also contribute to the burden of sub-optimal serum magnesium,^34^ as vitamin D can stimulate intestinal magnesium absorption.^35^

In Qatar, the prevalence of obesity is among the highest in the world.^36^ In our study, over 70% of the participants were overweight/obese. To the best of our knowledge, this is the first large study to examine the association between serum magnesium and adiposity measured by DEXA in the general population. Our major findings were consistent with previous studies on the association between magnesium status and obesity measured by BMI.^17^,^37–40^ In a 30-year longitudinal study of 5115 American adults, after adjusting for potential confounding factors, dietary magnesium intake is inversely associated with the incidence of obesity.^39^ Compared with the lowest quintile of magnesium intake, the highest quintile had a 51% (95% CI 40–60%) reduced risk of developing obesity. In the SUN (Seguimiento Universidad de Navarra) project, during a mean of 9.6 years follow-up of 14,057 adults dietary magnesium intake below 200 mg/d was associated with the risk of incident hypertension, stronger for overweight/obese participants.^41^ In a systematic review of 32 randomized clinical trials, magnesium supplementation (48–450 mg/d) resulted in a reduction in BMI of 0.21 kg/m^2^.^37^ The beneficial effect was mainly observed among those with magnesium deficiency and obesity at baseline. The inverse association between serum magnesium and metabolic syndrome in our study is also in line with another study conducted in the Chinese population.^42^

Several mechanisms may explain the association between magnesium and body adiposity. Firstly, magnesium has antioxidant effects, thus reducing levels of oxidative stress and inflammation.^43^ It is well known that inflammation increases the risk of obesity. In our study, comparing those with CRP above and below 6 mg/L, the difference in total fat mass was 9 kg. Secondly, evidence from a systematic review suggests that magnesium nutritional status is associated with depression.^44^ A high intake of magnesium is associated a lower risk of depression. Studies have shown that depression increases the risk of obesity.^21^ Thirdly, magnesium is related to sleep duration and quality. Short sleep duration and poor sleep quality increase the risk of obesity.^20^ Finally, an adequate magnesium intake can positively affect the composition of gut microbiota and inhibit fat deposition.^1^ In a clinical trial, a dietary supplement rich in bioactive magnesium and other trace elements significantly increases the gut microbial diversity in adult rats^45^.

Despite the above hypothesized mechanisms, we did not find any mediation effect via depression and sleep duration. In our study, the association between serum magnesium and fat mass was mainly observed among those without chronic conditions such as diabetes, hypertension, and depression. It could be due to the fact that the inflammation levels are already high among those with chronic conditions and high level of serum magnesium alone may not be able to reduce inflammation related adiposity. Another possible explanation is the use of medication for chronic diseases. For example, hypertension medications or diuretics can increase magnesium removal from the body. In a meta-analysis of eighteen RCTs involving 15,309 diabetic patients, Sodium-glucose cotransporter 2 (SGLT2) inhibitors significantly increases serum magnesium levels as compared with placebo.^46^

The high burden of short sleep duration deserves attention. Short sleep duration is related to multiple health outcomes including obesity and diabetes.^20^ In our study population, the beneficial effect of magnesium on adiposity was only observed among those with adequate sleep duration.

The study has several limitations. Firstly, the cross-sectional study design does not allow a causal relationship to be made. Secondly, the study is limited by the lack of information on participants’ disease history and the duration of the disease as some diseases may affect the level of magnesium, use of medications and magnesium supplement use. Finally, accurate estimation of magnesium intake was not possible as the FFQ does not provide information on portion sizes. The strengths of the study are that it has large sample size measured for DEXA and the ability to adjust for various confounding factors.

## Conclusion

Low serum magnesium concentrations were associated with higher body fat. Women are more predisposed to low serum magnesium concentrations. Further longitudinal studies and clinical trials are needed to verify whether dietary magnesium and supplemental magnesium may affect body adiposity in the Qatari population. Based on available evidence of the beneficial effects of adequate magnesium intake, it is recommended to encourage adults with obesity to consume magnesium-rich food and consider supplementation if needed.
